# Demography and Population Dynamics of Massive Coral Communities in Adjacent High Latitude Regions (United Arab Emirates)

**DOI:** 10.1371/journal.pone.0071049

**Published:** 2013-08-21

**Authors:** Kristi A. Foster, Greg Foster

**Affiliations:** Nova Southeastern University Oceanographic Center, Dania Beach, Florida, United States of America; University of Sydney, Australia

## Abstract

Individual massive coral colonies, primarily faviids and poritids, from three distinct assemblages within the southeastern Arabian Gulf and northwestern Gulf of Oman (United Arab Emirates) were studied from 2006–2009. Annual photographic censuses of approximately 2000 colonies were used to describe the demographics (size class frequencies, abundance, area cover) and population dynamics under “normal” environmental conditions. Size class transitions included growth, which occurred in 10–20% of the colonies, followed in decending order by partial mortality (3–16%), colony fission (<5%) and ramet fusion (<3%). Recruitment and whole colony mortality rates were low (<0.7 colonies/m^2^) with minimal interannual variation. Transition matrices indicated that the Arabian Gulf assemblages have declining growth rates (λ<1) whereas the massive coral population is stable (λ = 1) in the Gulf of Oman. Projection models indicated that (i) the Arabian Gulf population and area cover declines would be exacerbated under 10-year and 16-year disturbance scenarios as the vital rates do not allow for recovery to pre-disturbance levels during these timeframes, and (ii) the Gulf of Oman assemblage could return to its pre-disturbance area cover but its overall population size would not fully recover under the same scenarios.

## Introduction

Global climate change is predicted to increase the frequency, intensity and duration of disturbances that impact coral reefs (e.g. [1]–[4]). As coral communities have been shown to require 10–30 years to recover after a major disturbance (e.g. [5]–[8]), it is possible that taxa susceptible to environmental disturbances (i.e. branching and tabular acroporids and pocilloporids) diminish or become locally extirpated while the resistant taxa (i.e. massive poritids and faviids) would become the dominant reef builders. Under such circumstances, it will be the fates of the surviving massive corals that shape future coral communities in the southeastern Arabian Gulf, the northwestern Gulf of Oman, and, by extension, other similarly structured coral reefs (if high latitude communities are indeed the precursors to tropical coral reefs influenced by climate change [9]–[10]).

Coral communities in the territorial waters of the United Arab Emirates have recently been exposed to a series of natural disturbances that have had significant impacts on branching and tabular *Acropora* and *Pocillopora* spp. colonies. Elevated temperature anomalies in 1996, 1998 and 2002 were associated with the mass mortality of up to 99% of the acroporids in the southeastern Arabian Gulf (i.e. Abu Dhabi and Dubai) [11]–[13]. Cyclone Gonu damaged >50% of the acroporids in the northwestern Gulf of Oman (e.g. Fujairah) in 2007 [14] and was followed by a *Cochlodinium polykrikoides* harmful algal bloom (HAB) in 2008–09 which resulted in mass mortality of *Pocillopora damicornis* [14]–[16]. The aforementioned disturbances had lesser effects on massive coral populations, with greater than 75% survival of poritids and faviids during each event [11], [13]–[14]. Coral dominance in both regions has shifted from highly susceptible branching and tabular species to more resistant massive species. Whether these shifts are short-lived or persistent depends on many factors including (i) recruitment of new acroporids and pocilloporids from local surviving colonies and from remote larval sources [5], [9], (ii) the frequency of disturbance events; and (iii) recruitment, growth and survival of massive corals.

The objectives of this study are to (i) describe the demographics and dynamics of the massive coral communities in the southeastern Arabian Gulf and the northwestern Gulf of Oman, (ii) use the vital rates, based on temporal comparisons of individual colonies, to develop size class transition probability matrices, and (iii) project the population sizes and live coral area cover for these communities over the next 40 years.

## Methods

### Annual Surveys

**Figure 1:**
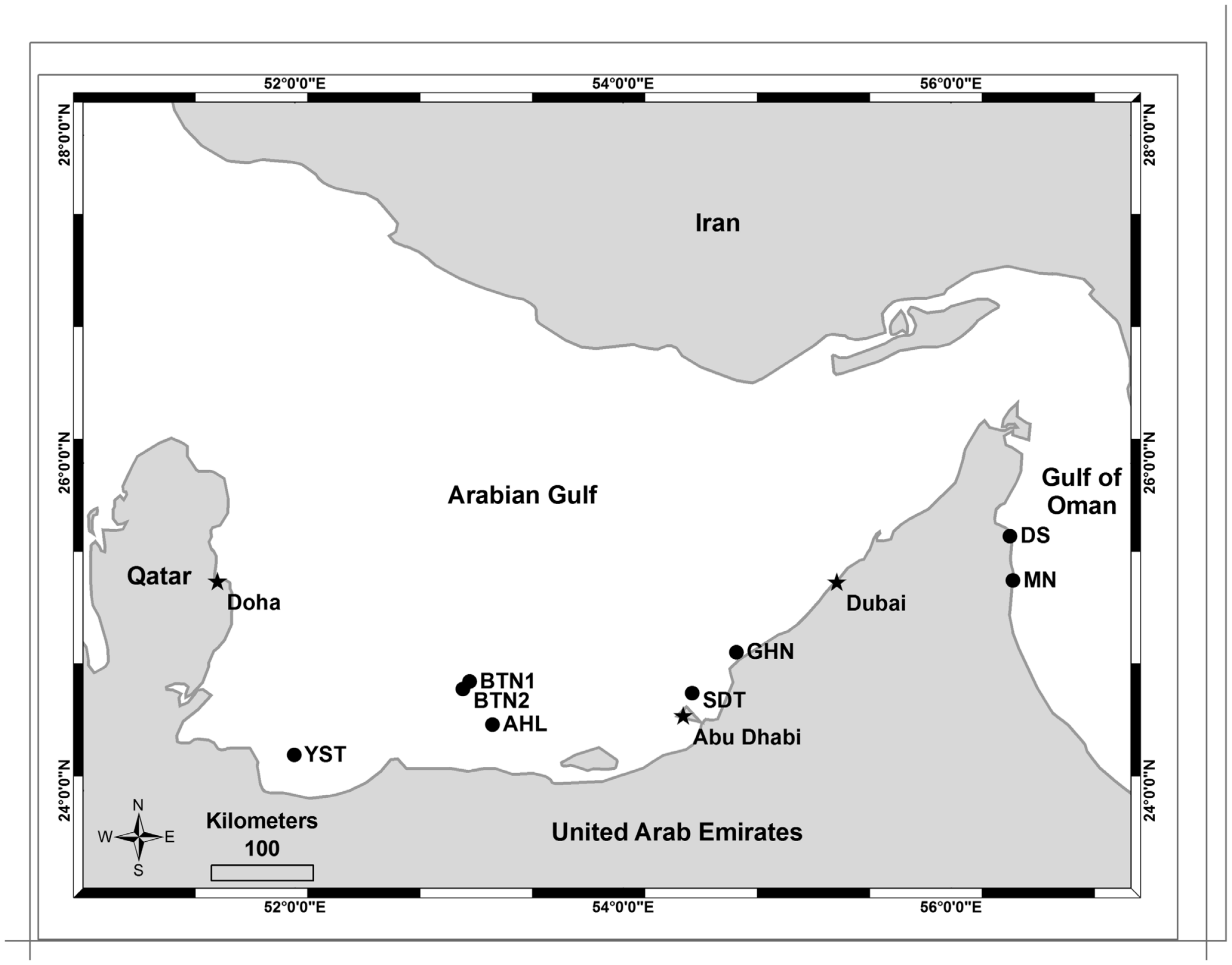
Map of southeastern Arabian Gulf and northwestern Gulf of Oman study areas. Monitoring station locations at Al Hiel (AHL), Bu Tinah (BTN1&2), Yasat (YST), Saadiyat (SDY), Ras Ghanada (GHN), Dibba South (DS), and Mirbah North (MN).

Hard coral populations were surveyed annually in the southeastern Arabian Gulf and northwestern Gulf of Oman between 2006 and 2009 (Figure 1, Table 1). Permission to conduct the surveys was granted by the respective regulatory agencies: (i) Environmental Agency – Abu Dhabi for all Arabian Gulf sites, (ii) Dibba-Fujairah Municipality and the Dibba Marine Centre of the Ministry of Environment and Water for the Dibba South site in the Gulf of Oman, and (iii) Fujairah Municipality for the Mirbah North site in the Gulf of Oman. Permanent monitoring stations were installed in order to allow for repetitive photographic surveys of benthic areas and specific coral colonies. Digital images were taken along three 10 m×1.5 m belt transects at depths <10 m within each monitoring station using a rigid photo-framer that oriented the camera at a fixed distance of 50 cm above the benthos. The 0.5 m×0.75 m base of the framer served as a border within each image to provide known dimensions for subsequent image analysis.

**Table 1:** Descriptions of repetitive monitoring sites in the southeastern Arabian Gulf and northwestern Gulf of Oman.

Station	Site Name	Depth (m)	Location	Region	Assemblage	Year(s)
YST	Yasat Ali	3.0–4.7	Island	SE Arabian Gulf	AG1	2006–09
BTN1	Bu Tinah North	1.8–3.6	Island	SE Arabian Gulf	AG1	2006–09
BTN2	Bu Tinah West*	2.0–3.5	Island	SE Arabian Gulf	AG1	2006–08
AHL	Al Hiel	2.6–4.2	Island	SE Arabian Gulf	AG1	2006–09
SDT	Saadiyat	5.7–7.2	Coastal	SE Arabian Gulf	AG2	2007–09
GHN	Ras Ghanada	7.6–8.5	Coastal	SE Arabian Gulf	AG2	2007–09
DS	Dibba South	6.7–8.1	Coastal	NW Gulf of Oman	GO	2007–09
MN	Mirbah North	4.5–6.9	Coastal	NW Gulf of Oman	GO	2007–09

* The Bu Tinah West monitoring station was damaged between 2007 and 2008, presumably as a result of winter storms; therefore, 2007–2008 and 2008–2009 temporal comparisons for Assemblage AG1 were based on the three remaining sites.

Images were joined into a single mosaic for each belt transect. A number was assigned to each massive coral that appeared as a whole colony within the photo mosaics. (Branching corals were also present in certain transects but were excluded from this study for which the focus was on the slower-growing, disturbance-resistant massive coral demographics. The status of the branching corals has been published elsewhere [17]). Each numbered coral was traced using the Area Analysis function in Coral Point Count (CPCe) [18], which calculated colony area cover (planar view). Transect data within each site were pooled to provide percent live coral cover and coral densities. Data processing for assemblage classification and ordination included (i) fourth root transformation for the production of a Bray-Curtis similarity matrix; (ii) agglomerative, hierarchical cluster analysis using group average sorting; and (iii) non-metric multi-dimensional scaling (nMDS). Non-parametric similarity of percentages (SIMPER) tests were performed to determine which taxa contributed the most to within-group similarities and among-group dissimilarities. All multivariate analyses were implemented using PRIMER software [19].

### Size Class Determination

Massive colonies were grouped into five size-dependent classifications (“SC”) (Table 2) based on area cover (where areas were assumed to be based on circular colonies with A = πr^2^). To determine the most appropriate groupings, size frequency distributions were compared for areas associated with radius increments of 1, 2, and 3 cm. The optimal, normally distributed size-dependent groupings were those based on radius increments of 2 cm. (Size classes based on radius increments of 1 cm and 3 cm were sub-optimal with frequency distributions skewed to the left and right, respectively.).

**Table 2:** Size-dependent classifications for massive coral colonies.

Size Class	Area Cover (cm^2^)	Est. Radius (cm)
1	<12.7	<2
2	12.7–50.2	2–4
3	50.3–113.0	4–6
4	113.1–201.1	6–8
5	>201.1	>8

Massive coral colonies were grouped into five size classes based on their measured area cover and estimated radii (assuming circular colonies, A = πr^2^).

### Transition Matrices

Size class transition matrices were developed for faviids, poritids and all massive corals (i.e. faviids, poritids, siderastreids, and dendrophylliids) in each of the regional assemblages. The use of five size classes resulted in 5×5 matrices in which each element represents the mean probability of moving from a starting size class or “state” (column) to ending size class or “fate” (row) [20]–[21]. The matrices include growth (G) to the next largest size class, size class stability (S) by remaining within the same group, or partial mortality (PM) to a smaller size class. Corals may also experience fission (the regression of a single colony into multiple smaller ramets) or fusion (i.e. two or more ramets grow together) [22]–[23]. In these cases, area coverages of each ramet set were pooled and compared to the size class for the respective parent colony which underwent fission or for the resulting fused colony. The probabilities of the fission and fusion transitions were added to the corresponding partial morality, size class stability or growth elements within the matrices. The resulting probability matrices, based on 4000+ individual size class transitions, were used to project the number of corals in each size class during year *t+1*, which equals the number in each size class at year *t* multiplied by the respective size class transition probabilities plus the mean number of corals which enter the population through recruitment (R) (Eq. 1). SC1 colonies that were visible within the belt transect images during a given year, but had not been visibile the previous survey, were recorded as recruits. (Image resolution was clear enough to identify colonies as small as 0.1 cm^2^ to genus; however, some of the smaller *Favia* and *Favites* colonies lacked the morphological characteristics that help to differentiate the species, so these taxa were pooled into the *Favia/Favites* group).
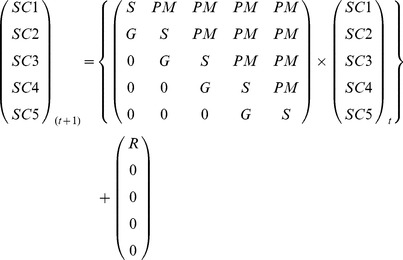
(1)


A 5×5 matrix has five eigenvalues, λ_i_, or solutions to the matrix. The dominant eigenvalue (i.e. the largest, positive eigenvalues that is a real number) is the growth rate of the size class-structure population [20], [24]: (i) for λ >1, the population is growing, (ii) for λ = 1, the population is stable, and (iii) for λ <1, the population is declining. The ratio of the dominant eigenvalue to the absolute value of the second largest eigenvalue, known as the damping ratio, provides the rate of convergence of the population toward a stable stage distribution (i.e. the larger the damping ratio, the quicker a population will return to its stable state after a disturbance) [20]–[21]. Sensitivities and elasticites, represented as surface plots, are measures of perturbation analyses that quantify the relative contribution of each vital rate to the population growth by adjusting each rate by a specific amount and by a specific proportion, respectively [20]–[21]. The dominant eigenvalues (i.e. population growth rates), stable size class distributions, sensitivities, and elasticities for the transition matrices were calculated using PopTools add-in for Excel [25].

### Projection Models

Projections were modeled through 2050 as idealized, best-case scenario forecasts of massive coral populations and live area cover [20]–[21]. Such projections assumed that (i) current parameters remain unchanged over time, (i.e. during normal, disturbance-free intervals); (ii) coral vital rates (e.g. growth, stability, fission, fusion, mortality) include the interactions among corals and other benthic organisms, responses to the surrounding environment and other factors that affect population structures; and (iii) the mean recruitment rates between 2006–2009 occur annually throughout the projection period.

For comparison, alternative disturbance scenarios were calculated for the assemblages whereby mass mortality events occur every 16 years (i.e. the midpoint between the historical 15–17 year disturbance intervals for the southeastern Arabian Gulf region [9]) and every 10 years (i.e. the timeframe between the two most recent disturbances which occurred in 2002 and 2012). Both disturbance internals were presumed to result in the death of 25% of the massive corals [11], [13] while the population dynamics for the surviving 75% of the corals remain unchanged.

## Results and Discussion

### Hard Coral Assemblages

**Figure 2:**
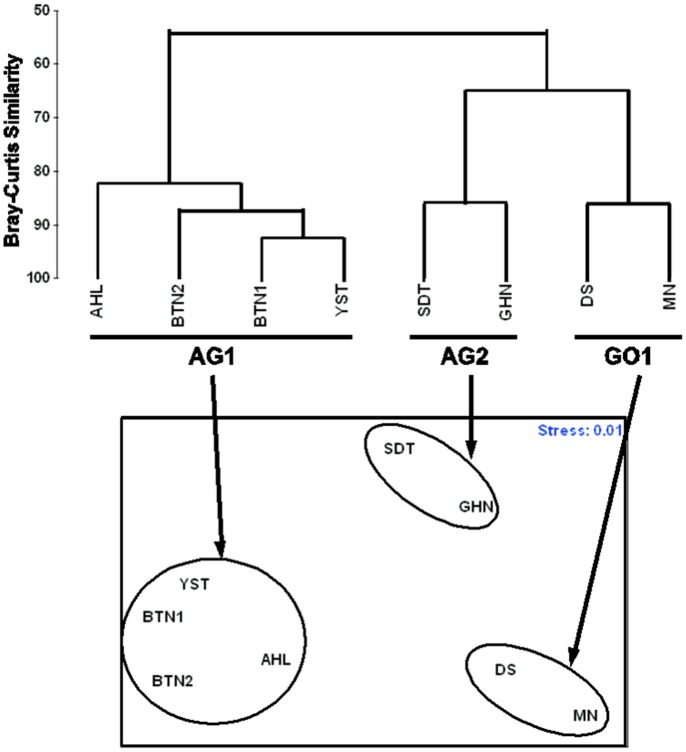
Coral assemblages by cluster analysis and multi-dimensional scaling. (upper) Bray-Curtis similarity cluster analysis depicting three assemblages; AG1, AG2 and GO1. (lower) MDS graphic representation with ovals around assemblages indentified by dendrogram. AG1 is comprised of Al Hiel (AHL), Bu Tinah (BTN1&2) and Yasat (YST). AG2 is comprised of Saadiyat (SDY) and Ras Ghanada (GHN). GO1 is comprised of Dibba South (DS) and Mirbah North (MN).

Cluster analysis differentiated three hard coral assemblages (designated AG1, AG2 and GO1), each with >80% between-site similarity (Figure 2). AG1 and AG2 are subsets of the southeastern Arabian Gulf sites whose hard coral populations were sparse and moderate populations, respectively, of *Porites harrisoni* and other massives. Assemblage GO1 consisted of two sites along the northwestern coast of the Gulf of Oman that were moderately populated by *Platygyra daedalea, Favia spp.* and other massive corals.

Site selections for this study were made haphazardly to include a cross-section of known coral community locations (i.e. frequently visited coastal sites as well as locations near offshore ranger stations) and independently of population demographics. However, it was not surprising that the Gulf of Oman sites comprised an assemblage separate from the Arabian Gulf sites. Exposure to salinity and seawater temperature extremes (i.e. ≥40 ppt and 14–36°C in the Arabian Gulf [26]–[27] compared to 36.5 ppt and 22–31°C [28]–[29] in the Gulf of Oman) has limited the number of species in the Arabian Gulf to approximately one-third of those found in the Gulf of Oman [11], [29]–[31]. In this study, only 10 of the 15 scleractinian genera recorded at the Gulf of Oman monitoring stations were also observed in the Arabian Gulf.

AG1 and AG2 were located in the southwest corner of the study area and near the Abu Dhabi coast, respectively (Figure 1). Further studies are needed to determine whether this constitutes a true west-east geographic gradient or if other factors contribute to the different community compositions (e.g. proximity to the prevailing surface current, coastal versus island dynamics). Prior surveys have characterized the coral communities near Dubai (approximately 115 km eastward of this study) into five well-separated assemblages [5], [12]. AG1 and AG2 were compositionally similar to the massive coral understories of two of these Dubai assemblages [17] which may suggest that these assemblages (and possible others) are distributed throughout the region and that the apparent geographic groupings of AG1 and AG2 were coincidental.

### Population Structures

#### AG1

**Figure 3:**
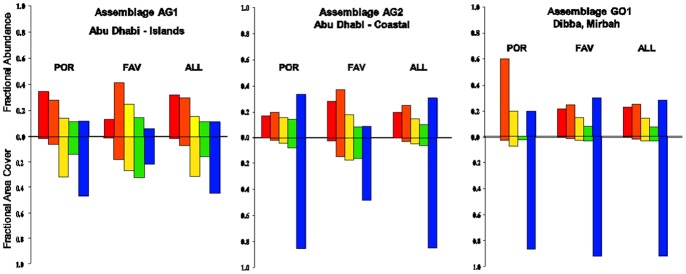
Size frequency distributions for massive corals by region. POR = poritids, FAV = faviids; ALL = all massive coral taxa Size Class color coding: SC1 = red, SC2 = orange, SC3 = yellow, SC4 = green, SC5 = blue.

A sparsely populated assemblage (7% area cover) dominated by *Porites harrisoni, Platygyra daedalea* and *Cyphastrea microphthalma* (Table 3). Mean coral density was 2.8 live colonies/m^2^, comprised primarily of size class (SC) 1–2 colonies (i.e. area cover ≤50.2 cm^2^) (Figure 3). Subordinate taxa included faviids (*Favia, Favites, Leptastrea* spp.), other poritid species (*P. solida, P. lutea*) and two *Siderastrea savignyana* colonies. Live acroporids were not observed within the vicinities of the monitoring stations; however, consolidated rubble indicated that acroporids had existed within these sites at one time.

**Table 3:** Taxa groups responsible for >90% within-group similarities and among-group dissimilarities based on SIMPER analysis.

Arabian Gulf 1 (AG1)			Groups: AG1/AG2		
Average similarity: 70.89	Cont. (%)	Cum. (%)	Average dissimilarity: 48.15	Cont. (%)	Cum. (%)
*Porites*	59.4	59.4	*Platygyra*	23.5	23.5
*Platygyra*	22.9	82.3	*Favia/Favites*	22.5	45.9
*Cyphastrea*	8.3	90.6	*Porites*	20.0	66.0
			*Turbinaria*	15.9	81.8
			*Cyphastrea*	8.3	90.2
**Arabian Gulf 2 (AG2)**			**Groups: AG1/GO1**		
Average similarity: 84.23			Average dissimilarity: 56.71		
*Porites*	40.5	40.5	*Platygyra*	35.4	35.4
*Platygyra*	28.5	69.0	*Favia/Favites*	21.8	57.2
*Favia/Favites*	21.1	90.1	*Porites*	16.9	74.2
			*Siderastrea*	9.6	83.8
			*Cyphastrea*	6.7	90.5
**Gulf of Oman (GO1)**			**Groups: AG2/GO1**		
Average similarity: 81.05			Average dissimilarity: 34.9		
*Platygyra*	52.9	52.9	*Porites*	41.9	41.9
*Favia/Favites*	29.0	81.9	*Turbinaria*	18.0	59.9
*Porites*	10.3	92.2	*Platygyra*	14.1	74.0
			*Siderastrea*	11.2	85.2
			*Coscinaria*	5.7	90.9

Cont. (%) is the percentage contributed by the respective taxa group to the (dis)similarity. Cum (%) is the cumulative percentage of (dis)similarity.

#### AG2

A moderately populated assemblage (32% area cover) dominated by *P. harrisoni*, *P. daedalea* and the *Favia/Favites* group (Table 3). Mean massive coral density was 12.7 live colonies/m^2^, consisting primarily of SC5 poritids (i.e. area covers >201.1 cm^2^) and SC1–2 faviids (Figure 3). Subordinate taxa included other faviids (*C. microphthalma, Leptastrea transversa*), other poritids (*P. solida, P. lutea, P. nodifera*) and other massive coral species (*S. savignyana*, *Coscinaraea columna*, *Turbinaria reniformis*). Acroporids were also observed within this assemblage, comprising <2.2% of the total benthic area cover, but these were excluded from this study as the focus was on the massive coral demographics. (The acroporids were subordinate to the massive corals in number and area cover and are likely to remain subordinate unless this assemblage experiences an extended disturbance-free period of >15 years, one or more recruitment pulses of >6 recruits per year, or both [17]).

#### GO1

A moderately populated assemblage (32% area cover) dominated by *P. daedalea,* the *Favia/Favites* group and mixed *Porites* spp. (Table 3). Mean massive coral density was 4.6 live colonies/m^2^, comprised primarily of SC5 *Platygyra* spp. and SC1–2 mixed faviids (Figure 3). Subordinate taxa included other faviids (*Cyphastrea, Leptastrea, Plesiastrea*), poritids (*Goniopora*) and siderastreids (*Coscinaraea, Psammacora, Pseudosiderastrea, Siderastrea*). Sporadic pocilloporids and acroporids were observed within this assemblage. The maximum branching coral cover (3.8%) was observed at the Mirbah North monitoring station in 2006 (i.e. prior to Cyclone Gonu) during a 12–40 year disturbance-free period [14] within which the massive corals established and retained dominance over the branching corals. Annual turnover of post-cyclone pocilloporid and acroporid recruits indicates that branching corals are likely to may remain subordinate to the massive corals in the near future [17].

#### Recruitment

Faviid and poritid recruit sizes ranged between 0.1 and 12.6 cm^2^, with a mean area cover of 4.4±3.2 cm^2^. First year recruits near Abu Dhabi and in the Gulf of Oman were approximately half the size of those recorded as juveniles/recruits in other regional studies (e.g. ≤4 cm max diameter in Dubai [5] and <5 cm diameter in the Red Sea [32]). Use of the broader juvenile/recruit grouping would have included SC1 and SC2 colonies herein; however, only 15% of the combined SC1 and SC2 size classes were first year recruits. A similar analysis of the SC1 colonies indicated that first year recruits comprised 32% of SC1 colonies, with the remainder being juveniles or small adults that exhibited size class stability (43%) or shrinkage from larger size classes (25%). Such results may aid future regional studies derive recruitment estimates from datasets that cannot differentiate first-year recruits from juveniles or small adults.

Recruit abundance ranged between 0.0 and 0.3 colonies/m^2^, with an exception of 0.70 faviids/m^2^ in 2008–09 within AG2 (Table 4). The mean winter seawater temperature in the southeastern Arabian Gulf was 1–2°C warmer in 2008–09 than in 2006–07 and 2007–08 [33], which perhaps contributed to the more favorable faviid recruit survival in the region. While seawater temperature has been reported as a significant factor related to gamete maturation and spawning in the Arabian Gulf [34], further investigations are needed to determine whether winter seawater temperatures impact the survival and growth of recently settled larvae into SC1 colonies the following year.

**Table 4:** Populations and sexual recruitment of faviids, poritids, and all massive corals.

	Faviids			Poritids			All Massives		
	06–07*	07–08	08–09	06–07*	07–08	08–09	06–07*	07–08	08–09
**AG1**									
Live colonies	68	68	63	455	296	284	523	365	348
Recruits	2	0	5	29	11	38	31	11	43
Live colonies/m^2^	0.4	0.5	0.5	2.5	2.2	2.1	2.9	2.7	2.6
Recruits/m^2^	<0.1	N/A	<0.14	0.2	0.1	0.3	0.2	0.1	0.3
**AG2**									
Live colonies		764	769		343	368		1128	1159
Recruits		1	63		7	23		8	88
Live colonies/m^2^		8.5	8.5		3.8	4.1		12.5	12.9
Recruits/m^2^		<0.1	0.7		<0.1	0.3		<0.1	1.0
**GO1**									
Live colonies		294	252		6	2		304	260
Recruits		3	6		0	3		3	9
Live colonies/m^2^		3.3	5.6		<0.1	<0.1		3.4	5.8
Recruits/m^2^		<0.1	<0.1		N/A	<0.1		<0.1	0.2

Assemblages: AG1– Arabian Gulf 1; AG2 = Arabian Gulf 2; GO1 = Gulf of Oman.

* = includes Bu Tinah West monitoring station.

In general, the numbers of recruits were comparable between assemblages in a given year, despite the population density in AG2 being approximately four times greater than in AG1 and GO1, suggesting that factors other than adult densities within the local populations are influencing recruitment success. For example, faviid recruit:adult ratios in 2009 were 1∶12 in both AG1 and AG2 despite a greater than tenfold difference in adult densities. In contrast, poritid recruit:adult ratios during 2009 were 1∶7 and 1∶16 for AG1 and AG2, respectively, although the adult densities were of the same order of magnitude. Recruit:adult ratios were considerably lower (1∶42) for faviids and higher (3∶2) for poritids in the Gulf of Oman compared to the Arabian Gulf. Further investigations are required to identify the spatial variation patterns, if any, and possible contributing factors.

#### Whole colony mortality

Whole colony mortality ranged between 0.0 and 0.6 colonies/m^2^, equivalent to ≤16% of the population (Table 5), demonstrating that minor levels of mortality occur as part of “normal” turnover in these populations (i.e. in the absence of a major disturbance) [22]). Whole colony mortality occurred most frequently in SC1 and SC2 corals (63% and 21% of all mortalities, respectively). In most cases, these colonies were no longer visible in subsequent years, indicating either removal from the substrate or overgrowth. The probability of mortality decreased with increasing colony size, a pattern that has been reported in other studies (e.g. [22]–[23], [35]–[38]).

**Table 5:** Whole colony mortalities.

	Faviids			Poritids			All Massives		
	06–07*	07–08	08–09	06–07*	07–08	08–09	06–07*	07–08	08–09
**AG1**									
Whole colony deaths deaths	4	6	10	65	45	31	69	52	42
Deaths/m^2^	<0.1	<0.1	<0.1	0.4	0.3	0.2	0.4	0.4	0.3
Percent Mortality (%)	5.6	8.8	15.9	14.3	15.2	10.9	13.2	14.2	12.1
**AG2**									
Whole colony deaths deaths		6	21		4	19		10	40
Deaths/m^2^		<0.1	0.2		<0.1	0.2		0.1	0.4
Percent Mortality (%)		0.8	2.7		1.2	5.2		0.9	3.4
**GO1**									
Whole colony deaths		1	27		0	1		1	29
Deaths/m^2^		<0.1	0.6		N/A	<0.1		<0.1	0.6
Percent Mortality (%)		0.3	10.7		N/A	50.0		0.3	11.2

Assemblages: AG1– Arabian Gulf 1; AG2 = Arabian Gulf 2; GO1 = Gulf of Oman,

* = includes Bu Tinah West; Percent Mortality is the percent of the population that experienced whole colony death.

The corals within the GO1 were exposed to a red tide event during the 2008–2009 sample period [14], yet whole colony death was similar to that for AG1 faviids and all massive corals which were not exposed to a similar disturbance. Such results indicate that additional studies are needed to determine the proportion of deaths attributable to a disturbance above and beyond normal population losses.

### Size Class Stability, Growth and Partial Mortality

Size class stability was the most likely fate for colonies; 45–65% of the colonies in AG1, 70–74% in AG2, and 70% in GO1 (faviids). The probability of size class stability increased with increasing colony size (Table 6), with mean annual probabilities ≥0.845 for SC5 colonies. A high proportion of size stability is not unexpected as it may take a colony several years to transition into a larger size class based on an annual growth rate of 1–2 cm for massive corals in this region [9].

**Table 6:** Size class stabilities (no size class transitiona).

	Faviids			Poritids			All Massives		
	06–07*	07–08	08–09	06–07*	07–08	08–09	06–07*	07–08	08–09
**AG1**									
SC1	7	1	2	67	13	10	74	14	12
SC2	11	6	3	54	30	29	65	36	32
SC3	12	18	14	26	20	17	38	38	31
SC4	11	8	6	22	17	20	33	25	26
SC5	3	4	9	62	54	82	65	58	91
Total	44	37	34	231	134	158	275	171	192
% of population	64.7%	54.4%	54.0%	50.8%	45.3%	55.6%	52.6%	46.8%	55.2%
Colonies/m^2^	0.2	0.3	0.3	1.3	1.0	1.2	1.5	1.3	1.4
**AG2**									
SC1		112	97		10	10		123	107
SC2		209	227		24	27		240	259
SC3		92	102		25	16		119	120
SC4		38	42		16	24		54	68
SC5		86	78		180	193		268	273
Total		537	546		255	270		804	827
% of population		70.3%	71.0%		74.3%	73.4%		71.3%	71.4%
Colonies/m^2^		6.0	6.1		2.8	3.0		8.9	9.2
**GO1**									
SC1		15	6		0	0		15	6
SC2		39	36		2	0		43	38
SC3		21	18		1	1		23	19
SC4		9	5		0	0		9	5
SC5		123	97		2	0		125	97
Total		207	162		5	1		215	165
% of population		70.4%	64.3%		83.3%	50.0%		70.7%	63.5%
Colonies/m^2^		2.3	3.6		<0.1	<0.1		2.4	3.7

Assemblages: AG1– Arabian Gulf 1; AG2 = Arabian Gulf 2; GO1 = Gulf of Oman.

SC = Size class;

* = includes Bu Tinah West.

Growth was the second most likely transition with 17–24% of the colonies in AG1, 12–15% in AG2, and 9–21% in GO1 (faviids) moving into the next larger size class (Table 7). The mean annual probability of growth increased with increasing size class for AG1 and AG2 poritids. No discernible trends were observed for faviids in any of the assemblages. It is interesting to note that faviid growth continued to occur with the GO1 population despite prolonged exposure to the red tide between 2008 and 2009 [14].

**Table 7:** Growth profiles (postive size class transitions).

	Faviids			Poritids			All Massives		
	06–07*	07–08	08–09	06–07*	07–08	08–09	06–07*	07–08	08–09
**AG1**									
SC1 = > SC2	1	1	2	33	14	6	34	15	8
SC2 = > SC3	9	6	3	19	14	20	28	20	23
SC3 = > SC4	2	3	6	16	22	13	18	25	19
SC4 = > SC5	2	5	2	11	22	18	13	27	20
Total	14	15	13	79	72	57	93	87	70
% of population	20.6%	22.1%	20.6%	17.4%	24.3%	20.1%	17.8%	23.8%	20.1%
Colonies/m^2^	<0.1	0.1	0.1	0.4	0.5	0.4	0.5	0.6	0.5
**AG2**									
SC1 = > SC2		59	47		5	3		65	50
SC2 = > SC3		27	37		14	11		41	49
SC3 = > SC4		17	10		14	11		34	22
SC4 = > SC5		9	7		15	19		25	26
Total		112	101		48	44		165	147
% of population		14.7%	13.1%		14.0%	12.0%		14.6%	12.7%
Colonies/m^2^		1.2	1.1		0.5	0.5		1.8	1.6
**GO1**									
SC1 = > SC2		15	4		0	0		15	4
SC2 = > SC3		15	5		0	0		15	5
SC3 = > SC4		17	9		1	0		18	10
SC4 = > SC5		14	5		0	0		14	5
Total		61	23		1	0		62	24
% of population		20.7%	9.1%		16.7%	0.0%		20.4%	9.2%
Colonies/m^2^		0.7	0.5		<0.1	N/A		0.697	0.5

Values exclude colonies and ramets that underwent fusion.

Assemblages: AG1– Arabian Gulf 1; AG2 = Arabian Gulf 2; GO1 = Gulf of Oman.

SC = Size class;

* = includes Bu Tinah West.

A slightly smaller percentage of all colonies, 3–16%, experienced partial mortality (i.e. shrinkage of live tissue area, unfragmented by bare skeleton) and transitioned into smaller size classes (Table 8). In the Arabian Gulf, >86% of the colonies that underwent partial mortality regressed only one size class (SC5 = > SC4, SC4 = > SC3, SC3 = > SC2, SC2 = > SC1) rather than multiple size classes, which provides a baseline for negative size class transitions under “normal” environmental conditions. In the Gulf of Oman, faviid partial mortality doubled between 2008 and 2009; however, additional studies are needed to determine whether this difference was due, in part or entirely, to the red tide or if it was within the range of interannual variability.

**Table 8:** Partial mortalities (negative size class transitions).

	Faviids			Poritids			All Massives		
	06–07*	07–08	08–09	06–07*	07–08	08–09	06–07*	07–08	08–09
**AG1**									
SC5 = > SC4	1	1	0	6	5	6	7	6	6
SC5 = > SC3	0	0	0	4	1	1	4	1	1
SC5 = > SC2	0	0	0	0	1	0	0	1	0
SC5 = > SC1	0	0	0	0	0	0	0	0	0
SC4 = > SC3	0	1	0	10	5	6	10	6	6
SC4 = > SC2	0	1	1	0	0	1	0	1	2
SC4 = > SC1	0	0	0	0	0	0	0	0	0
SC3 = > SC2	1	4	1	6	8	2	7	12	3
SC3 = > SC1	0	0	1	3	1	1	3	1	2
SC2 = > SC1	0	0	0	26	6	6	26	6	6
Total	2	7	3	55	27	23	57	34	26
% of population	2.9%	10.3%	4.8%	12.1%	9.1%	8.1%	10.9%	9.3%	7.5%
Colonies/m^2^	<0.1	<0.1	<0.1	0.3	0.2	0.2	0.3	0.3	0.2
**AG2**									
SC5 = > SC4		10	10		20	13		30	25
SC5 = > SC3		2	3		0	1		2	4
SC5 = > SC2		1	3		2	0		3	4
SC5 = > SC1		0	1		1	1		1	2
SC4 = > SC3		18	15		6	7		26	22
SC4 = > SC2		0	3		2	4		2	7
SC4 = > SC1		0	1		0	0		0	2
SC3 = > SC2		38	21		4	5		44	27
SC3 = > SC1		1	2		0	1		1	3
SC2 = > SC1		39	42		1	3		40	49
Total		109	101		36	35		149	145
% of population		14.3%	13.1%		10.5%	9.5%		13.2%	12.5%
Colonies/m^2^		1.2	1.1		0.4	0.4		1.7	1.6
**GO1**									
SC5 = > SC4		7	6		0	0		7	6
SC5 = > SC3		0	4		0	0		0	4
SC5 = > SC2		0	4		0	0		0	4
SC5 = > SC1		0	0		0	0		0	0
SC4 = > SC3		5	11		0	0		5	11
SC4 = > SC2		4	1		0	0		4	1
SC4 = > SC1		0	0		0	0		0	0
SC3 = > SC2		6	6		0	0		6	7
SC3 = > SC1		0	2		0	0		0	2
SC2 = > SC1		3	6		0	0		4	6
Total		25	40		0	0		26	41
% of population		8.5%	15.9%		0.0%	0.0%		8.6%	15.8%
Colonies/m^2^		0.3	0.9		N/A	N/A		0.3	0.9

Values exclude colonies that underwent fission.

Assemblages: AG1– Arabian Gulf 1; AG2 = Arabian Gulf 2; GO1 = Gulf of Oman.

SC = Size class;

* = includes Bu Tinah West.

### Fission and Fusion

Fission and fusion played minor roles in the dynamics of the AG1, AG2 and GO1 populations, with respective mean annual probabilities of 0.00–0.06 and 0.0–0.03 (Tables 9–10). Similar fission probabilities for other massive and foliaceous species were reported in Jamaica (0.02–0.10; [23]) and in Australia (0.01–0.06; [22]), indicating that low rates of fission occur among subtropical and tropical coral communities even in the absence of environmental stresses such as those associated with seawater temperature extremes, hurricanes and other natural disturbances. Low probabilities of fusion, in some circumstances, may be attributed to the rates of tissue reconnection/growth which are currently understudied. Certainly the extent of tissue loss during fission and the distance between ramets will impact whether fusion in a subsequent year is possible. Several years of growth may be required before ramets are capable of reconnecting during which barriers (e.g. algal growth on exposed skeleton) may prevent fusion. Other hindrances to fusion include additional shrinkage and mortality of the ramets since previously damaged corals have an increased likelihood of further damage [22].

**Table 9:** Fission – Parent colonies that underwent fission and mean number of ramets generated.

	Faviids			Poritids			All Massives		
	06–07*	07–08	08–09	06–07*	07–08	08–09	06–07*	07–08	08–09
**AG1**									
SC1 parents	0	0	0	1	0	0	1	0	0
SC2 parents	1	1	0	9	2	2	10	3	2
SC3 parents	2	2	2	1	1	2	3	3	4
SC4 parents	1	0	1	1	4	1	2	4	2
SC5 parents	0	0	0	1	5	2	1	5	2
% of population	5.9%	4.4%	4.8%	2.9%	4.1%	2.5%	3.2%	4.1%	2.9%
Colony fission/m^2^	<0.1	<0.1	<0.1	<0.1	<0.1	<0.1	<0.1	0.1	<0.1
Mean # of ramets	2.0	3.0	2.3	2.1	2.5	2.3	2.2	2.5	2.3
**AG2**									
SC1 parents		0	0		0	0		0	0
SC2 parents		2	0		1	0		3	3
SC3 parents		2	1		3	0		5	6
SC4 parents		2	1		1	1		3	4
SC5 parents		2	1		15	1		17	19
% of population		1.0%	0.4%		5.8%	0.5%		2.5%	1.6%
Colony fission/m^2^		<0.1	<0.1		0.2	<0.1		0.3	0.2
Mean # of ramets		2.1	2.0		2.3	2.0		2.2	2.2
**GO1**									
SC1 parents		0	0		0	0		0	0
SC2 parents		1	4		0	0		1	4
SC3 parents		1	0		0	0		1	0
SC4 parents		1	0		0	0		1	0
SC5 parents		7	13		0	0		7	13
% of population		3.4%	6.7%		0.0%	0.0%		3.3%	5.0%
Colony fission/m^2^		0.1	0.4		N/A	N/A		0.1	0.4
Mean # of ramets		2.1	2.7		0.0	0.0		2.1	2.7

Assemblages: AG1– Arabian Gulf 1; AG2 = Arabian Gulf 2; GO1 = Gulf of Oman.

SC = Size class;

* = includes Bu Tinah West.

**Table 10:** Fusion –Fused colonies and mean number of ramets that fuse together.

	Faviids			Poritids			All Massives		
	06–07*	07–08	08–09	06–07*	07–08	08–09	06–07*	07–08	08–09
**AG1**									
SC1	0	0	0	2	0	0	2	0	0
SC2	0	0	0	1	1	0	1	1	0
SC3	0	0	0	8	1	1	8	1	1
SC4	0	0	0	0	0	2	0	0	2
SC5	0	0	0	0	3	5	0	3	5
% of population	0	0	0	2.4%	1.7%	2.8%	2.1%	1.4%	2.3%
Colonies/m^2^	0	0	0	<0.1	<0.1	<0.1	<0.1	<0.1	<0.1
Mean # of ramets	0	0	0	2.5	2.0	2.4	2.5	2.0	2.4
**AG2**									
SC1		1	0		0	1		1	1
SC2		0	5		1	1		1	6
SC3		3	2		3	1		6	3
SC4		1	3		1	1		2	4
SC5		3	3		3	8		6	11
% of population		1.0%	1.7%		2.3%	3.3%		1.4%	2.2%
Colonies/m^2^		<0.1	0.1		<0.1	0.1		0.2	0.3
Mean # of ramets		2.3	2.2		2.1	2.3		2.2	2,2
**GO1**									
SC1		0	0		0	0		0	0
SC2		1	0		0	0		1	0
SC3		0	2		0	0		0	2
SC4		0	0		0	0		0	0
SC5		0	2		0	0		0	2
% of population		0.3%	1.6%		0.0%	0.0%		0.3%	1.5%
Colonies/m^2^		<0.1	<0.1		N/A	N/A		<0.1	<0.1
Mean # of ramets		2.0	2.3		0.0	0.0		2.0	2.3

Assemblages: AG1– Arabian Gulf 1; AG2 = Arabian Gulf 2; GO1 = Gulf of Oman.

SC = Size class;

* = includes Bu Tinah West.

On average, 2–3 ramets were generated when a parent colony underwent fission. The majority (79–89%) of the pooled ramet surface areas were in the same size classes as their respective parent colonies whereas 11–21% of the fissions resulted in transitions to smaller size classes. Similarly, 2–3 ramets grew together to generate a fused colony. The majority (66–76%) of the fused AG1 and AG2 colonies were in the same size classes as their respectively pooled ramets whereas 24–33% of the fusions resulted in transitions to larger size classes. All GO1 faviid fusions were recorded as size class stability transitions.

#### Size class transition matrices

Mean transition probability matrices were developed for faviids, poritids and all massive corals (Table 11). Little information has previously been published regarding the life histories of the massive coral species within the Arabian Gulf [9] and the Gulf of Oman. The vital rates presented herein may provide actual data for other predictive models that would otherwise utilize estimations of recruitment, mortality, or growth.

**Table 11:** Transition probability matrices.

		Faviids						Poritids						All Massive Corals			
**AG1**																	
	SC1	SC2	SC3	SC4	SC5		SC1	SC2	SC3	SC4	SC5		SC1	SC2	SC3	SC4	SC5
SC1	0.412	0.000	0.025	0.000	0.000	SC1	0.330	0.136	0.033	0.000	0.000	SC1	0.333	0.114	0.031	0.000	0.000
SC2	0.230	0.413	0.081	0.056	0.000	SC2	0.223	0.490	0.106	0.014	0.005	SC2	0.224	0.475	0.100	0.022	0.005
SC3	0	0.345	0.699	0.056	0.000	SC3	0	0.244	0.470	0.162	0.026	SC3	0	0.260	0.540	0.137	0.025
SC4	0	0	0.151	0.663	0.150	SC4	0	0	0.337	0.452	0.080	SC4	0	0	0.279	0.499	0.083
SC5	0	0	0	0.225	0.850	SC5	0	0	0	0.359	0.876	SC5	0	0	0	0.331	0.875
*** s***	0.642	0.758	0.956	1.000	1.000	***s***	0.553	0.870	0.946	0.987	0.987	***s***	0.557	0.849	0.950	0.989	0.988
*** d***	0.358	0.242	0.044	0.000	0.000	***d***	0.447	0.130	0.054	0.013	0.013	***d***	0.443	0.151	0.050	0.011	0.012
**AG2**																	
	SC1	SC2	SC3	SC4	SC5		SC1	SC2	SC3	SC4	SC5		SC1	SC2	SC3	SC4	SC5
SC1	0.623	0.139	0.010	0.006	0.005	SC1	0.486	0.045	0.015	0.000	0.005	SC1	0.606	0.129	0.011	0.007	0.004
SC2	0.315	0.746	0.205	0.022	0.021	SC2	0.201	0.600	0.118	0.062	0.005	SC2	0.303	0.727	0.188	0.037	0.011
SC3	0	0.109	0.684	0.247	0.026	SC3	0	0.297	0.519	0.141	0.002	SC3	0	0.131	0.642	0.208	0.010
SC4	0	0	0.094	0.597	0.103	SC4	0	0	0.320	0.423	0.080	SC4	0	0	0.148	0.520	0.090
SC5	0	0	0	0.120	0.845	SC5	0	0	0	0.365	0.905	SC5	0	0	0	0.219	0.883
*** s***	0.938	0.994	0.993	0.992	1.000	***s***	0.687	0.942	0.972	0.991	0.998	***s***	0.909	0.987	0.989	0.991	0.998
*** d***	0.062	0.006	0.007	0.008	0.000	***d***	0.313	0.058	0.028	0.008	0.002	***d***	0.091	0.013	0.011	0.009	0.002
**GO1**																	
	SC1	SC2	SC3	SC4	SC5								SC1	SC2	SC3	SC4	SC5
SC1	0.484	0.053	0.001	0.000	0.000							SC1	0.484	0.065	0.000	0.000	0.000
SC2	0.484	0.684	0.136	0.125	0.000							SC2	0.484	0.694	0.128	0.125	0.000
SC3	0	0.263	0.477	0.156	0.000							SC3	0	0.242	0.489	0.156	0.000
SC4	0	0	0.386	0.281	0.054							SC4	0	0	0.383	0.281	0.053
SC5	0	0	0	0.438	0.946							SC5	0	0	0	0.438	0.947
*** s***	0.968	1.000	1.000	1.000	1.000							***s***	0.968	1.000	1.000	1.000	1.000
*** d***	0.032	0.000	0.000	0.000	0.000							***d***	0.032	0.000	0.000	0.000	0.000

Assemblages: AG1– Arabian Gulf 1; AG2 = Arabian Gulf 2; GO1 = Gulf of Oman. SC = Size Class. Columns depict starting state, rows depict ending fate. *s* = probability of survival within the respective size class, equal to the sum of probabilities in each column*; d* = probability of whole colony death (1-s).

Although seemingly short, the 2–4 years of repetitive monitoring used to generate the size class transition probability matrices for AG1 and AG2 is comparable to other vital rate studies for corals, gorgonians and sponges [22]–[23], [39]–[44]. Ideally, annual data collection would continue in order to determine whether the coral communities follow predictable cycles or whether irregular patterns are the norm.

GO1 was exposed to a prolonged red tide event that persisted between August 2008 and May 2009 [14]. The impacts of this disturbance on vital rates (e.g. possible increased whole colony death and partial mortality, decreased growth and size class stability) were not independently tested. Because the focus of this study was on the fate of massive corals under “normal” environmental conditions, transition matrices and projections for GO1 were based on surveys in 2007 and 2008 only. These results are included herein as a first published report of vital rates for the massive corals in this region but should be considered as preliminary.

The stable size class distributions (i.e. the eigenvectors associated with the dominant eigenvalues), dominant eigenvalues and damping ratios were determined for each assemblage (Table 12). The dominant eigenvalues (λ) for AG1 and AG2 were <1, which result in gradual population decay, whereas the GO1 eigenvalue (λ = 1) indicates a stable population [20]. The damping ratios were 1.1–1.3, indicating that faviids and poritids approach asymptotic behavior (stability) at similar rates among the assemblages (i.e. similar resilience/recovery following a disturbance) [45].

**Table 12:** Stable size class distributions, dominant eigenvalues and damping ratios.

	AG1				AG2				GO1	
	FAV	POR	ALL		FAV	POR	ALL		FAV	ALL
**Stable SC Distributions**				**Stable SC Distributions**				**Stable SC Distributions**		
SC1	0.005	0.015	0.014	SC1	0.183	0.016	0.120	SC1	0.008	0.010
SC2	0.049	0.043	0.045	SC2	0.453	0.069	0.318	SC2	0.078	0.081
SC3	0.120	0.111	0.122	SC3	0.229	0.092	0.199	SC3	0.066	0.066
SC4	0.304	0.173	0.184	SC4	0.072	0.149	0.113	SC4	0.093	0.091
SC5	0.523	0.658	0.636	SC5	0.062	0.674	0.250	SC5	0.755	0.753
**Dominant Eigenvalue**				**Dominant Eigenvalue**				**Dominant Eigenvalue**		
λ	0.981	0.970	0.971	*λ*	0.984	0.986	0.981	λ	1.000	1.000
Real or complex	Real	Real	Real	Real or complex	Real	Real	Real	Real or complex	Real	Real
**Damping Ratio**				**Damping Ratio**				**Damping Ratio**		
λ_1_/|λ_2_|	1.2	1.3	1.3	λ_1_/|λ_2_|	1.1	1.2	1.1	λ_1_/|λ_2_|	1.1	1.1

Assemblages: AG1– Arabian Gulf 1; AG2 = Arabian Gulf 2; GO1 = Gulf of Oman. Coral taxa: FAV = faviids; POR = poritids; ALL – all massive coral taxa. SC = Size Class.

### Sensitivities and Elasticities

**Figure 4:**
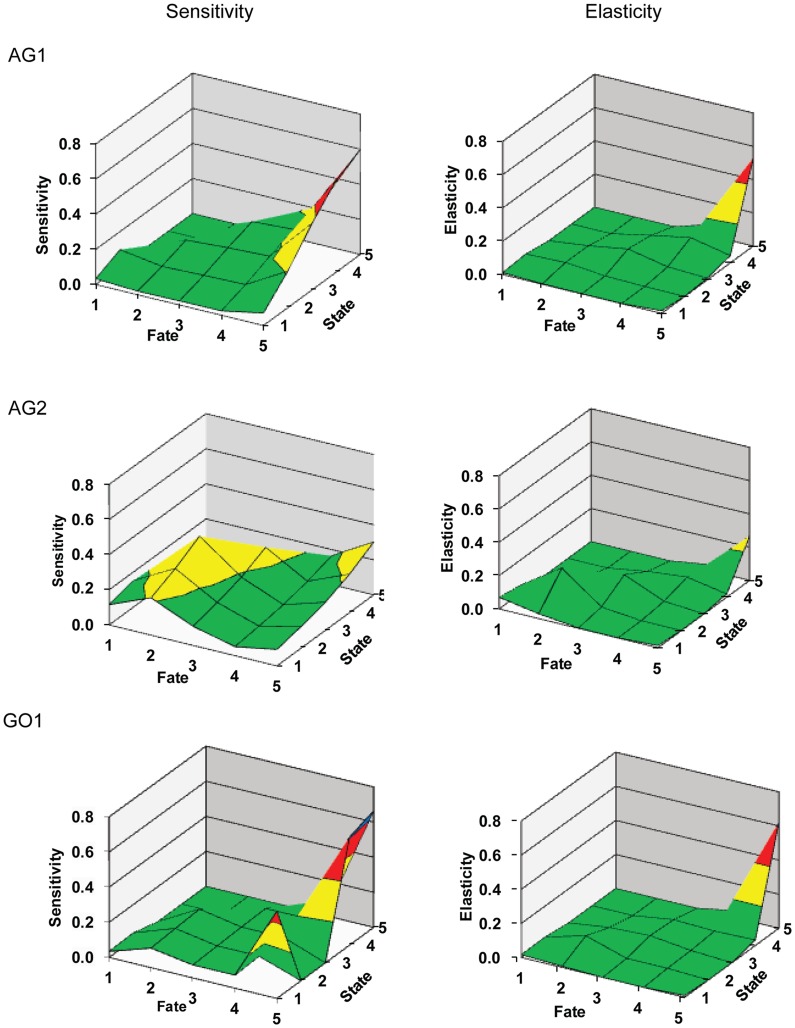
Sensitivity and elasticity surface plots for all massive corals by region. Fate and state axes represent transitions between size classes 1–5. Vertical axes represent the sensitivity and elasticity of the respective population growth rates, λ, to perturbation analyses. Sensitivity/Elasticity color coding: 0.0–0.2 = green, 0.2–0.4 = yellow, 0.4–0.6 = red, 0.6–0.8 = blue.

Senstivities and elasticities are measures of perturbation analyses that quantify the relative contribution of each vital rate to the population growth by adjusting each rate by a specific amount and by a specific proportion, respectively [20]–[21]. All sensivity and elasticity matrices, displayed graphically as surface plots (Figure 4), indicated that the dominant eigenvalues, λ, were most affected by changes in the upper right corners of the transition matrices which correspond to the stability of SC5 colonies, partial mortality of SC5 into SC4, and growth of SC4 into SC5. Sensitivities in AG2 were affected, in decreasing order, by the growth of SC2, SC2 stability, and growth of SC3, due to the large population of smaller faviids within this assemblage. Sensitivies in GO1 were affected by recruitment of SC1 colonies into the population.

### Population Projections

**Figure 5:**
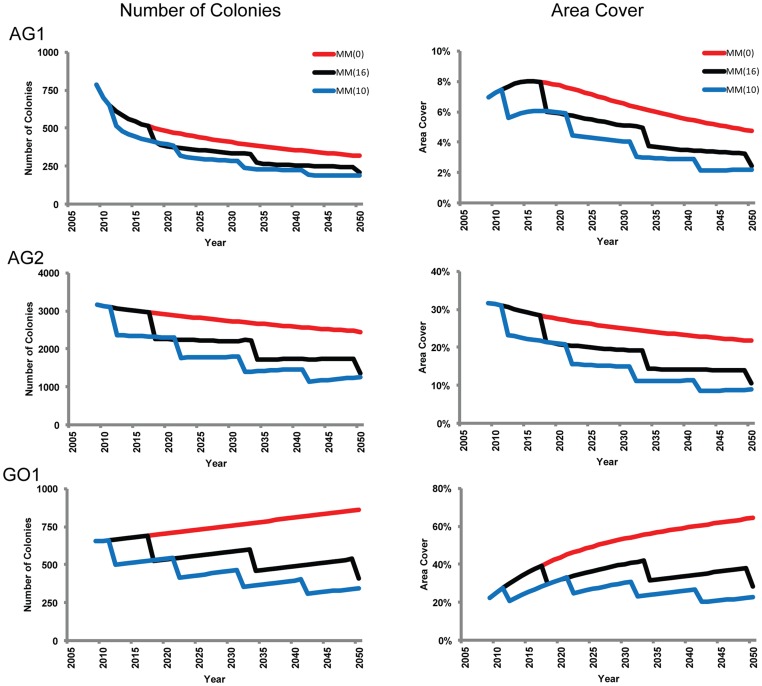
Population and area cover projections through 2050 for all massive corals. Red MM(0) lines represesent optimal, disturbance-free projections (zero mass mortality events). Black MM(16) and blue MM(10) lines represent mass mortality every 16 years and 10 years, respectively, with 25% loss of massive corals during each disturbance.

The size class transition matrices were used to project populations through 2050 (Figure 5). The following projections are idealized and are not expected to occur but, rather, are shown as best case, disturbance-free scenarios:

1. AG1 corals are not projected to reach a stable size class distribution due to the continual change in the number and proportion of the SC4–SC5 colonies. The number of colonies decline by 60% through 2050 due to the mean annual probabilities of mortality exceeding those for recruitment. Despite the decrease in colony density, area cover (7.0% in 2009) will temporarily increase to a maximum of 8.0% through 2014–2017, due to the temporary increase in the number of SC5 colonies, then gradually decrease to 4.8% by 2050.
2. AG2 faviids are projected to reach stable distributions, dominated by SC2 colonies, around 2015–2020. AG2 poritids are not projected to reach a stable size class distribution, due primarily to the changing number and proportion of SC5 colonies. The number of faviids increase by 3% while the poritids decrease by 45%; the net result is a 23% decrease in the massive coral population and a gradual decline from 32% to 22% area cover over the projection period.
3. The distribution of SC1–SC4 faviids in GO1 are projected to stabilize around 2020; however the number of SC5 colonies will continue to gradually increase through 2050. (The poritid community was too small for projections.) The GO1 projected faviid area cover approaches 60% under idealized conditions; however, this is likely an overestimation resulting from transition probabilities that were based on a two-year data set.

The most recent 10-year and the historical 16-year [9] disturbance intervals for this region were projected through 2050 (Figure 5) with the following results:

1. The 10-year and 16-year intervals are insufficient to allow AG1 and AG2 massives to recover from the population and area cover losses associated with each disburbance.
2. GO1 populations approach but fall short of the predisturbance levels within the 10-year and 16-year scenarios; however, borh invervals are sufficient to return to the respective pre-disturbance area covers.

The fates of all three assemblages depend heavily on the continued health of the SC5 colonies. With declining populations in both Arabian Gulf assemblages (plus the low area cover in AG1) during normal environmental conditions, these populations are at risk of collapse should a large proportion of the SC5 colonies become compromised due to natural or anthropogenic stresses (e.g. mass mortality, disease outbreaks, coastal development). Current recruitment levels are insufficient to replace losses associated with major disturbance events (e.g. up to 25% loss of massive corals [11], [13]) as demonstrated in the 10- and 16-year disturbance frequency scenarios (Figure 5).

### Conclusions

Little information pertaining to hard coral vital rates within the Arabian Gulf and the Gulf of Oman has been published to date. This study documents the population dynamics during “normal” environmental conditions which may be used as baseline comparisons when conducting coral community health surveys, when reporting the effects of disturbance events (e.g. temperature anomalies, cyclonces, red tides, disease outbreaks) or when developing predictive ecological models for this region. Important findings related to the massive corals in the UAE are summarized as follows:

1. First year recruits have maximum radii ≤2 cm; however, only 32% of the colonies within this size range are recruits whereas the remainder is comprised of juveniles and small adults. Mean annual recruit abundance is typically low (≤0.7 recruits/m^2^), exclusive of possible recruitment pulses which were not recorded during this study. Recruitment success does not appear to be heavily influenced by adult densities within the local population.
2. Whole colony mortality and partial mortality (i.e. shrinkage into a smaller size class) may each effect up to 16% of the population in a given year as part of “normal” turnover.
3. Colonies may take several years to transition into a larger size class due to the slow growth rate for massive corals; only 9–24% of the population experiences growth whereas 45–74% maintains size class stability in a given year.
4. Fission and fusion play minor roles in the population dynamics, effecting 0–6% and 0–3% of the colonies, respectively.

The size class transition probability matrices developed in this study indicate that the Arabian Gulf massive coral assemblages have negative population growth rates (λ <1) under “normal” environmental conditions. Projection models show that 10-year and 16-year disturbance intervals further exacerbate the population declines. It is, therefore, critical that these assemblages be protected, to whatever extent possible, from disturbances that are detrimental to their demographics or population dynamics (e.g. disturbances resulting in decreased recruitment, the loss of SC5 colonies, or increased whole colony or partial mortality). This is especially true in locations where poritids and faviids take the place of acroporids and pocilloporids as the dominant reef builders (i.e. following temperature anomalies, cyclones, and red tides to which the branching and tabular colonies are more susceptible) because it will be the massive taxa that sustain the coral communities and their associated biota during such recovery periods.
